# Autopercepción de las habilidades para la cesación del tabaquismo entre los residentes de cardiología en Argentina

**DOI:** 10.47487/apcyccv.v4i3.304

**Published:** 2023-09-30

**Authors:** Alan R. Sigal, Braian Abel Cardinali-Re, Lucas Campana, Pilar Lopez-Santi, Pablo Iomini, Celeste A. Zanoni, Mariana Salcerini, Leandro Pozzer, Manuel Traghetti, Laura Pulido, Daniel José Piñeiro, Andrés Rosende, Sebastián Garcia-Zamora, Consejo Argentino de Residentes de Cardiología (CONAREC)

**Affiliations:** 1 Consejo Argentino de Residentes de Cardiología (CONAREC), Buenos Aires, Argentina. Consejo Argentino de Residentes de Cardiología (CONAREC) Buenos Aires Argentina; 2 Servicio de Neumonología, Hospital Italiano de Rosario, Rosario, Argentina. Servicio de Neumonología Hospital Italiano de Rosario Rosario Argentina; 3 Facultad de Medicina, Universidad de Buenos Aires (UBA), Buenos Aires, Argentina. Universidad de Buenos Aires Facultad de Medicina Universidad de Buenos Aires (UBA) Buenos Aires Argentina; 4 Organización Panamericana de la Salud (OPS), Washington DC, Estados Unidos. Organización Panamericana de la Salud (OPS) Washington DC Estados Unidos; 5 Servicio de Cardiología, Sanatorio Delta, Rosario, Argentina. Servicio de Cardiología Sanatorio Delta Rosario Argentina

**Keywords:** Tabaquismo, Cese del Uso de Tabaco, Cardiología, Educación Médica, Argentina, Tobacco Use Disorder, Tobacco Use Cessation, Cardiology, Education, Medical, Argentina

## Abstract

**Objetivo.:**

Evaluar la autopercepción de los residentes de cardiología en Argentina con respecto a sus habilidades para que sus pacientes dejen de fumar, así como sus opiniones sobre sus conocimientos y habilidades en esta área.

**Materiales y métodos:**

. Estudio transversal a partir de los datos secundarios de un estudio realizado en cinco países de Latinoamérica y España, centrándose en la información proporcionada por los residentes de cardiología en Argentina. Las variables discretas se expresaron como mediana y rango intercuartílico, las variables categóricas se expresaron como porcentajes, y se analizaron mediante la prueba de chi cuadrado o la prueba exacta de Fisher, según la frecuencia relativa de los valores esperados.

**Resultados:**

. Participaron 447 residentes; el 87,5% proporcionaba rutinariamente consejos breves para dejar de fumar y el 11,6% utilizaba cuestionarios validados para evaluar el grado de adicción. Además, el 32,1% manifestó que prescribía tratamiento farmacológico, pero el 53,1% solo estaba familiarizado con un solo fármaco. Cuando se les preguntó sobre su autopercepción para lograr que sus pacientes dejen de fumar, la mediana de respuesta fue 5 (escala del 1 al 10); solo el 13,7% respondió con un puntaje de 8 o más.

**Conclusiones:**

. El presente estudio sugiere que los residentes de cardiología en Argentina reconocen la importancia de realizar intervenciones para dejar de fumar, pero una alta proporción de ellos no se sienten capacitados para hacerlo.

## Abstract (en)

**Objective.:**

To evaluate the self-perception of cardiology residents in Argentina regarding their abilities to help their patients stop smoking, as well as their opinions about their knowledge and skills in this area.

**Materials and methods.:**

A cross-sectional study was carried out using secondary data from a study carried out in five Latin American countries and Spain, focusing on the information provided by cardiology residents in Argentina. Discrete variables were expressed as median and interquartile range, and categorical variables were expressed as percentages, and were analyzed using the chi-square test or Fisher’s exact test, depending on the relative frequency of the expected values.

**Results.:**

447 residents participated; 87.5% routinely provided brief advice to quit smoking, and 11.6% used validated questionnaires to assess the degree of addiction. Furthermore, 32.1% stated that they prescribed pharmacological treatment, but 53.1% were only familiar with a single drug. When asked about their self-perception of getting their patients to stop smoking, the median response was 5 (scale from 1 to 10); only 13.7% responded with a score of 8 or more.

**Conclusions.:**

The present study suggests that cardiology residents in Argentina recognize the importance of carrying out smoking cessation interventions, but a high proportion of them do not feel qualified to do so.

## Introducción

El tabaquismo es uno de los principales factores de riesgo modificables a nivel mundial. Se le atribuyen directamente 8 000 000 muertes anualmente, con una tendencia creciente para los próximos años ^1^. En Argentina se producen alrededor de 44 000 muertes al año por enfermedades relacionadas con la adicción al tabaco ^2^. Esta adicción se asocia directamente con el desarrollo de enfermedades cardiovasculares, cerebrovascular, neoplasias en diferentes órganos y enfermedades respiratorias, entre otras afecciones ^3^^,^^4^. Las personas que experimentan un evento adverso relacionado con el consumo de tabaco están especialmente dispuestas a dejar esta adicción. Este vínculo directo entre el tabaquismo y las enfermedades cardiovasculares sitúa a los médicos cardiólogos en una posición privilegiada para ayudar a sus pacientes a dejar de fumar.

Un estudio realizado en cinco países de Latinoamérica y España ^5^, acerca de la preparación de los residentes de cardiología sobre cesación tabáquica, encontró que la mayoría de ellos considera que la cesación tabáquica es un tema relevante, pero un porcentaje importante no se siente suficientemente preparado para abordarlo y gran parte de quienes no emplean terapia farmacológica aducen falta de familiaridad con los tratamientos. Estos hallazgos subrayan la necesidad de mejorar la formación en cesación tabáquica para los futuros cardiólogos, lo que podría tener un impacto significativo en la prevención de enfermedades cardiovasculares.

Sin embargo, parece haber un marcado desequilibrio entre la magnitud del problema del consumo de tabaco, los beneficios de abandonar esta adicción y la formación que reciben los profesionales de salud en Argentina. Por este motivo, el objetivo del presente estudio fue evaluar la autopercepción de los residentes de cardiología en Argentina con respecto a sus habilidades para que sus pacientes dejen de fumar, así como sus opiniones sobre sus conocimientos y habilidades en esta área.

## Materiales y métodos

### Diseño y población de estudio

Entre noviembre de 2018 y enero de 2019 se realizó una encuesta voluntaria y anónima entre residentes, concurrentes, becarios y jefes de residentes de cardiología en centros públicos y privados de seis países de habla hispana: Argentina, Chile, España, México, Paraguay y Uruguay ^5^. El cuestionario se distribuyó utilizando un método de muestreo no probabilístico tipo «bola de nieve» y se difundió mediante correo electrónico y mensajes de texto a todos los residentes de cardiología en estos países. El presente estudio de corte transversal se basa en un análisis de datos secundarios de dicha encuesta, centrándose en la información proporcionada por los residentes de cardiología en Argentina.

### Variables del estudio

Se utilizó un cuestionario diseñado específicamente para el estudio, que incluyó 26 preguntas divididas en cinco secciones: a) Prioridad y evaluación de la cesación tabáquica; b) Intervenciones de cesación tabáquica; c) Uso de fármacos para la cesación tabáquica, d) Formación y autopercepción de los participantes sobre sus habilidades en cesación tabáquica, y e) Datos demográficos y características de los participantes

La plataforma empleada para administrar la encuesta a los participantes fue Google Forms®.

Se consideró como exfumadores a los participantes que manifestaron haber fumado en el pasado, pero habían dejado de hacerlo al menos 12 meses antes de responder a la encuesta.

Para las preguntas en las que se les solicitó a los participantes que indicaran la importancia que asignaban a un tema específico, se utilizó una escala lineal del 1 al 10, donde 10 representaba la máxima importancia y 1, la mínima.

### Análisis estadístico

Las variables discretas se expresaron como mediana y rango intercuartílico, las variables categóricas se expresaron como porcentajes, y se analizaron mediante la prueba de chi cuadrado o la prueba exacta de Fisher, según la frecuencia relativa de los valores esperados. 

Se asumió un nivel de significancia del 5% para todos los análisis estadísticos. Todos los análisis estadísticos se realizaron en dos colas. Se utilizó Stata versión 13.0 (Stata Corp., College Station, TX, EE. UU.) para llevar a cabo los análisis.

### Aspectos éticos

La encuesta incluyó un preámbulo en el que se detallaron los objetivos del proyecto y se enfatizó que la participación era voluntaria. Del mismo modo, se explicitó a los encuestados que los datos serían tratados de manera anónima y de acuerdo con las leyes nacionales e internacionales de protección de datos. Para evitar cualquier sesgo en las respuestas, se omitieron los datos de identificación, como la edad, el género, el año de formación y el centro de formación. Finalmente, se indicó que los encuestados aceptaban voluntariamente su participación y otorgaban su consentimiento de manera tácita al responder la encuesta. El proyecto fue aprobado por el Comité Asesor de CONAREC.

## Resultados

Un total de 447 médicos residentes, concurrentes, becarios y jefes de residentes de Argentina participaron en la encuesta. Aproximadamente un tercio de ellos llevaba a cabo su formación en diversas localidades de la provincia de Buenos Aires, mientras que casi la cuarta parte lo hacía en la Ciudad Autónoma de Buenos Aires (CABA). La Tabla 1 proporciona detalles sobre la provincia de residencia de todos los participantes. Del grupo encuestado, el 72,0% nunca había fumado, el 13,1% eran fumadores activos y el 14,9% eran exfumadores.

**Tabla 1:** Cantidad de participantes por provincia

Provincia	n (447)	Porcentaje
Provincia de Buenos Aires	145	32,4
Ciudad Autónoma de Buenos Aires	106	23,7
Santa Fe	44	9,8
Córdoba	31	6,9
Mendoza	30	6,7
Chaco	16	3,6
Corrientes	13	2,9
Tucumán	9	2,0
Salta	9	2,0
Neuquén	8	1,8
Misiones	8	1,8
Entre Ríos	7	1,6
Formosa	6	1,3
San Juan	6	1,3
La Rioja	3	0,7
Jujuy	3	0,7
Catamarca	2	0,4
Chubut	1	0,2

Cuando se les preguntó sobre la importancia que atribuían a la cesación tabáquica, la mediana de la respuesta fue 10 (rango intercuartílico [RIC] 8-10); solo 28 participantes (6,2%) puntuaron esta pregunta con un valor inferior a 7. El 87,5% de los encuestados afirmó que aconsejaba rutinariamente a sus pacientes fumadores que dejaran de fumar. En cuanto a la frecuencia con la que evaluaban el grado de adicción de los pacientes, el 38,7% dijo hacerlo siempre; el 27,7% lo hacía frecuentemente, y el 11,0% que nunca lo hacía. Entre los encuestados que afirmaron evaluar el grado de adicción de los pacientes al menos ocasionalmente, la forma más habitual de hacerlo fue de forma subjetiva o «sin ningún método específico». Solo el 11,6% de los participantes indicó utilizar un cuestionario validado para este fin.

**Figura 1:**
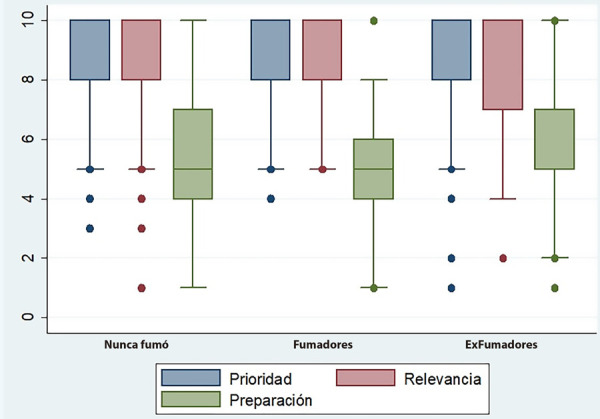
Intervenciones no farmacológicas aplicadas por los residentes de cardiología para el cese tabáquico de sus pacientes

El 41,6% de los encuestados expresó que realizaba intervenciones no farmacológicas para ayudar a sus pacientes a dejar de fumar. Sin embargo, la estrategia más frecuente entre los residentes encuestados fue derivar a los pacientes a un colega experto en cesación tabáquica como única intervención realizada por ellos (57,8%) **(**Figura 1**)**. No obstante, solo el 43,4% de los participantes tenía especialistas en cesación tabáquica en su centro.

Cuando se les preguntó sobre las intervenciones farmacológicas para la cesación tabáquica, el 32,1% manifestó que prescribían algún tratamiento para ayudar a sus pacientes en este proceso. El 53,1% de los participantes manifestó estar familiarizados con un solo tratamiento para dejar de fumar, siendo la terapia de reemplazo de nicotina la estrategia farmacológica más utilizada (31,3%), seguida del uso de bupropión (10,2%) y vareniclina (4,1%). El 22,4% de los encuestados dijo estar familiarizado con el empleo de dos estrategias farmacológicas para la cesación tabáquica, mientras que el 22,5% sabía cómo utilizar 3 o 4 grupos farmacológicos. Además, el 7,5% de los participantes utilizaban benzodiacepinas con este fin.

Entre aquellos que manifestaron utilizar tratamiento farmacológico para la cesación tabáquica, el 54,1% lo prescribían durante la internación, el 23,3% lo indicaban al momento del alta hospitalaria y el 22,6% en el control ambulatorio.

La mayoría de los encuestados que no utilizaban tratamiento farmacológico para la cesación tabáquica manifestaron que esto se debía a que no estaban familiarizados con este (59,7%). Entre los restantes, un 28,3% expresó que no tenía acceso a estos fármacos en su centro asistencial, o consideraba que sus pacientes tendrían dificultades para acceder a ellos debido al costo del tratamiento. Finalmente, el 4,3% no empleaba estas estrategias por temor a los efectos adversos o incremento en los eventos cardiovasculares, mientras que el 7,7% restante adujo otros motivos, como la negativa de sus superiores a esta prescripción.

El 45,9% de los encuestados afirmó haber recibido formación en cesación tabáquica a través de clases, cursos presenciales o en línea, discusiones bibliográficas, ateneos o rotaciones breves en consultorios especializados. En lo que respecta al grado de autopercepción sobre su preparación para lograr que una persona fumadora deje de fumar, la mediana de respuesta fue 5 (RIC 4-7), solo el 13,7% respondió a esta pregunta con una puntación igual o superior a 8.

**Figura 2:**
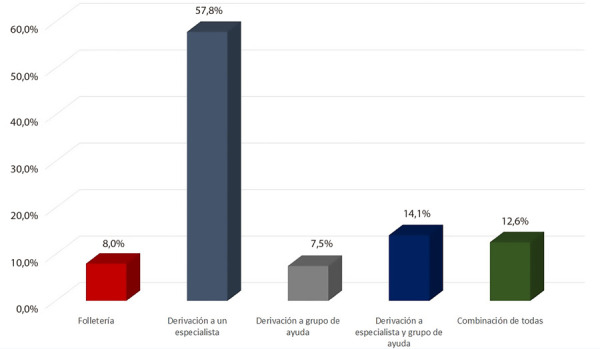
Prioridad, relevancia y preparación en cesación tabáquica entre residentes según su adicción al tabaco.

Finalmente, se exploró la relación entre la opinión de los participantes sobre la relevancia de este tema para su práctica diaria, la prioridad que le asignaban en su formación, su autopercepción sobre el grado de preparación para abordar esta problemática (empleando una escala lineal con puntuación de 1 a 10) y su condición frente al consumo de tabaco. No se encontraron diferencias entre los encuestados que nunca fumaron, aquellos que eran fumadores y los exfumadores en lo que respecta a la prioridad asignada a este tema (p=0,443) **(**Figura 2**)**. Lo mismo se observó al preguntar por la autopercepción en cuanto a su formación en este tema (p=0,470). Sin embargo, los participantes exfumadores consideraron esta temática menos relevante que aquellos que nunca fumaron y los fumadores actuales (8,58 ±1,8 comparado con 9,13 ±1,3 y 9,24 ±1,1, p=0,008).

## Discusión

El presente estudio tiene varios resultados importantes. En primer lugar, la mayoría de los residentes de cardiología en Argentina considera que la cesación tabáquica es un tema muy relevante para su práctica diaria. Además, nueve de cada diez residentes que participaron de la encuesta informaron que aconsejaban a sus pacientes fumadores a dejar de fumar. La intervención no farmacológica más empleada por los residentes de cardiología consistía en derivar a sus pacientes a otro especialista para recibir apoyo para dejar de fumar. Sin embargo, alrededor de un tercio residentes de cardiología expresó que prescribía tratamiento farmacológico para lograr la cesación tabáquica de sus pacientes. Finalmente, más de la mitad de los participantes en la encuesta expresó no sentirse capacitado para asistir a sus pacientes fumadores en el proceso de dejar de fumar. Estos resultados resaltan la necesidad de una mayor capacitación y apoyo en la cesación tabáquica para los profesionales de la cardiología en Argentina.

La relación entre el tabaquismo y múltiples problemas de salud ha sido establecida hace más de medio siglo; sin embargo, continúa siendo un problema sanitario mundial ^3^^,^^4^. Al mismo tiempo, la falta de formación en este tópico parece ser algo frecuente entre profesionales de la salud ^5^^-^^7^. Así, un estudio en terapistas respiratorios de Estados Unidos encontró que más de la mitad de los centros no ofrecía formación específica sobre cese del tabaquismo ^7^. Un hallazgo similar a lo observado en nuestro país, donde el 54,1% de los residentes de cardiología manifestó no haber recibido ninguna capacitación en este tema.

Esta falta de formación también se observa entre estudiantes de Medicina. Richmond *et al*. llevaron a cabo dos encuestas, con diez años de diferencia, en facultades de Medicina de más de 100 países, con el objetivo de valorar cuántas de ellas incluían materias o módulos específicos sobre cesación tabáquica ^8^^,^^9^. En la segunda edición del año 2009, los autores encontraron que los módulos específicos se incrementaron del 11 al 27%, mientras que 77% de las instituciones incluían la problemática del tabaquismo como parte de otras materias, comparado con un 40% en la edición de 1998 ^8^^,^^9^. En esta línea, existe evidencia de que los estudiantes de medicina que reciben formación específica sobre cesación tabáquica son capaces de implementarla en su práctica asistencial, al tiempo de que se perciben más seguros y eficaces para ayudar a personas con este problema ^10^, igual a lo que ocurre con los médicos titulados ^11^.

Sin embargo, la formación es solo un aspecto de un abordaje exitoso para el cese del tabaquismo. Barua *et al*. detallan que, además de la educación en cesación tabáquica, es necesario asignar roles específicos a cada profesional que entra en contacto con personas tabaquistas ^12^. Este hecho resulta crucial, ya que, en nuestro estudio, casi 3 de cada 5 encuestados derivaban a sus pacientes a un consultorio especializado como única intervención, en tanto que menos de la mitad de los participantes contaban con dichos especialistas en su centro. Esto pone de manifiesto que existen numerosas oportunidades perdidas para brindar consejería breve a pacientes fumadores, acción que debería ser llevada adelante por todos los profesionales de la salud, dado que no requiere de un entrenamiento prolongado o complejo ^12^^,^^13^. Así, tanto posicionamientos de expertos ^12^^-^^15^ como estudios específicos ^16^^,^^17^, dan cuenta de que programas de entrenamientos breves, de solo 2-3 horas, pueden ser suficientes para mejorar las habilidades de médicos de distintas especialidades para ayudar a sus pacientes a dejar de fumar ^18^^,^^19^. Incluso el personal no médico puede desempeñar un papel significativo en la cesación tabáquica ^20^.

Es importante reconocer que nuestro estudio tiene limitaciones que merecen ser consideradas a la hora de interpretar sus hallazgos. En primer lugar, dado que no se dispone del número exacto de cardiólogos en formación en Argentina, y debido al tipo de muestreo utilizado, no fue posible estimar la tasa de respuestas de la encuesta. Esto introduce sesgos de selección y de respuesta, y limita la validez externa de los resultados. Sin embargo, la proporción de residentes que participaron representa al menos la mitad de los cardiólogos en formación en Argentina, lo cual puede considerarse una muestra importante. En segundo lugar, no se dispone de los datos detallados de los participantes, como tampoco el dato de los centros donde llevaban adelante su formación. Muy probablemente algunas de estas características, como el año de residencia o ciertos aspectos particulares de los centros formadores, influencian el conocimiento de los encuestados sobre este tema. Sin embargo, también es cierto que la posibilidad de ser identificados podría haber sesgado las respuestas de los participantes, y este fue el motivo para omitir esta información. Además, esta encuesta no tiene la capacidad de valorar habilidades como la empatía y la escucha activa, ejes de la terapia para la cesación tabáquica. Sin embargo, a pesar de las limitaciones antes enunciadas, sería el primer estudio multicéntrico que evaluó las prácticas en cesación tabáquica de los residentes de cardiología en Argentina.

En conclusión, los hallazgos sugieren que la mayoría de los residentes de cardiología reconocen la importancia de la formación en cesación tabáquica durante su entrenamiento. Aunque muchos intentan ayudar a sus pacientes tabaquistas, derivar a otros profesionales fue la estrategia más utilizada. Además, más de la mitad de los participantes expresó sentirse inseguro sobre sus habilidades para abordar esta adicción. Consideramos necesario profundizar en el conocimiento de esta problemática, para reducir la disparidad en la atención y mejorar las intervenciones para personas con adicción al tabaco.
